# A windable and stretchable three-dimensional all-inorganic membrane for efficient oil/water separation

**DOI:** 10.1038/s41598-017-16402-5

**Published:** 2017-11-22

**Authors:** Kui Wang, Dong Suk Han, Wubulikasimu Yiming, Said Ahzi, Ahmed Abdel-Wahab, Zhaoyang Liu

**Affiliations:** 1 Qatar Environment and Energy Research Institute (QEERI), Hamad Bin Khalifa University (HBKU), Qatar Foundation, PO Box 5825 Doha, Qatar; 2 Key Laboratory of Traffic Safety on Track of Ministry of Education, School of Traffic and Transportation Engineering, Central South University, Changsha, 410075 Hunan China; 3 Chemical Engineering Program, Texas A&M University at Qatar, Education City, PO Box 23874, Doha, Qatar

## Abstract

There is strong interest in windable and stretchable membranes to meet the technological demands of practical water treatments. Oil/water separating membranes of this type is still significantly underdeveloped. Here, we reported a windable and stretchable membrane with three-dimensional structure for efficient oil/water separation. This membrane is made of ZnO nanorods arrays conformally grown on woven carbon microfibers. This three-dimensional architecture endows the fabricated membrane with highly windable and stretchable properties, at the same time ensures ZnO nanorods fully exposed outwards on the membrane surface. Due to its superior hydrophilicity and oleophobicity of ZnO nanorods, this all-inorganic membrane exhibits outstanding antifouling property, with the foulants on membrane surfaces easily removed by simple physical cleaning without chemicals. The membrane can effectively separate both oil/saline-water mixtures and oil-in-water emulsions, solely driven by gravity, with extremely high permeation flux of 20933.4 L m^−2^ h^−1^ and high separation efficiency over 99%.

## Introduction

Huge amounts of oily wastewaters are produced daily from the industries of oil production, oil refineries, petrochemical plants, chemical plants, as well as oil spill accidents. Under regulation, the oils in these oily wastewaters need to be removed to meet the stringent standard of discharge and reuse^1^. The advanced technologies to efficiently and economically remove the oils are in high demand. Compared with conventional technologies including gravity separation, air flotation and hydrocyclone, membrane separation is considered as a prominent technology in oil/water separation, thanks to its high separation efficiency, small foot print and easy operation. There have been great research interests in developing advanced membrane for oil/water separation^2–4^.

The rapid advancement in nanomaterials and nanotechnology over the past decade has presented new opportunity to scientific research in all fields. A series of advanced membranes with nanostructures and enhanced separation performance have been developed^5^. Among these advanced membranes, superwetting (superhydrophilic and under-water superoleophobic) membranes with nanostructured surface or ultrathin separation layers with nanometer-scale thickness are the most widely studied^6,7^. Due to their unique nanostructures, super-wetting membranes are capable of alleviating oil-fouling issues while exhibiting very high permeation flux and excellent separation efficiencies during oil/water separation^8^. However, none of these membranes reach commercial application level, due to their lack of windable and stretchable properties, which are critical important under hash operation condition for oil/water separation. The common practice of commercial membranes in water industry is in spiral wound module, which represents a big portion for practical applications, due to the small footprint and easy operation^9^. And meanwhile commercial membranes are expected to have advanced feature of stretchability in which the membranes are able to accommodate high mechanical stress and still maintain their functions.

Here, we demonstrated a novel all-inorganic oil-water separation membrane prepared by ZnO nanowires conformal grown on carbon cloths via low temperature hydrothermal reaction. The obtained ZnO nanowires/carbon cloth membrane showed outstanding superhydrophilicity, and underwater superoleophobicity. These excellent properties allowed the membrane to separate both oil-in-water emulsions and oil-in-saline-water mixtures by gravity-driven process with high permeation flux and high rejection efficiency. In addition, the prepared membranes showed high flexibility and mechanical strength at different temperatures indicating their great potential for practical application under extreme conditions.

## Results and Discussions

**Figure 1:**
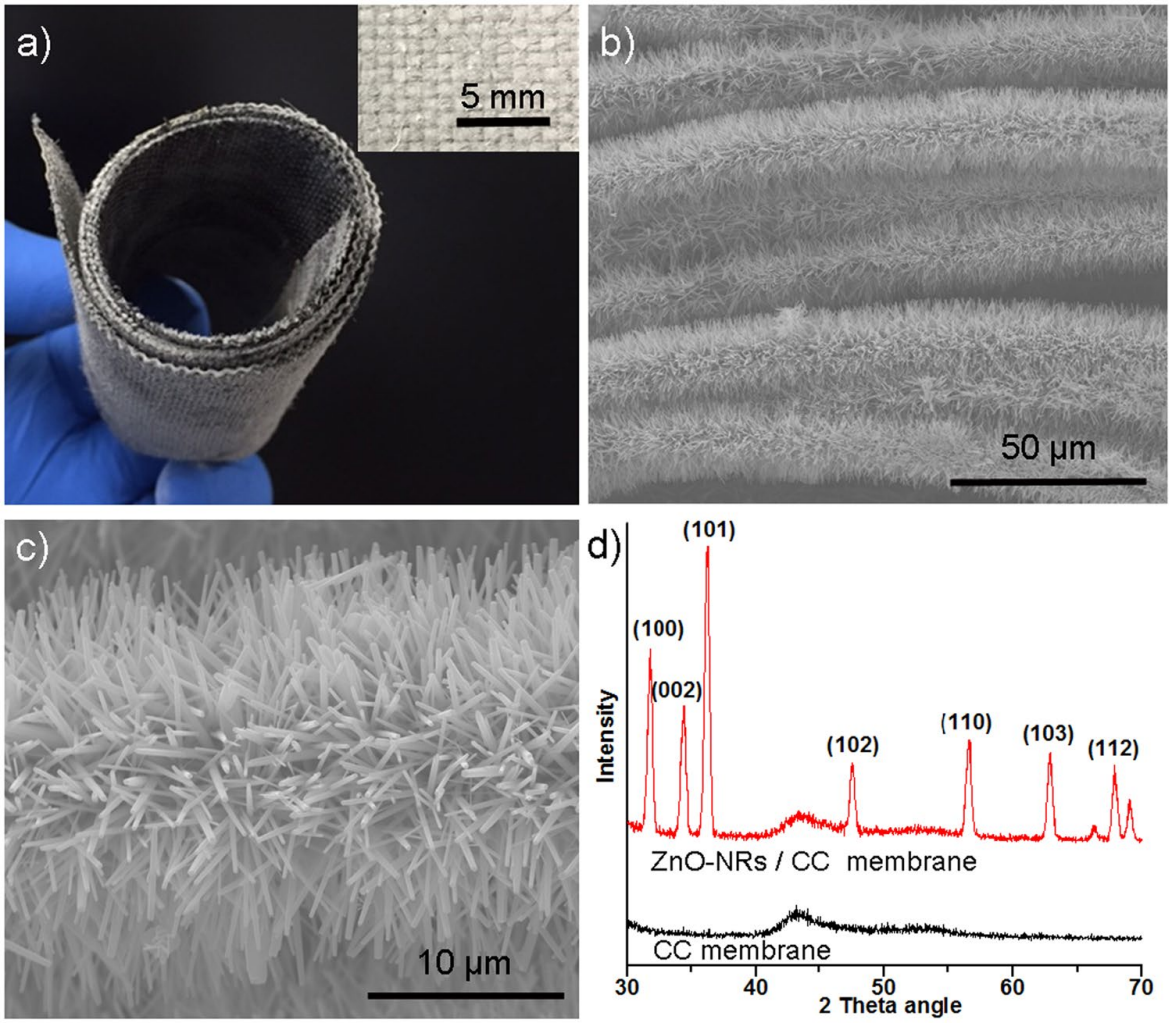
Structure characterization of ZnO-NRs/CC membrane with a hydrothermal reaction time of 24 h for growing ZnO-NRs on CC membrane. (**a**) Digital image of the dried flexible rolled-up membrane, the inset is a top-view of the dried as-prepared membrane. (**b**) SEM image of the membrane. (**c**) SEM image of a single carbon fiber with the growth of the ZnO-NRs. (**d**) XRD patterns of the ZnO-NRs/CC membrane compared with the original CC membrane, confirming the formation of the wurtzite structure of the ZnO for the ZnO-NRs/CC membranes.

The ZnO-NRs/CC membranes were prepared by growing the ZnO-NRs on carbon cloths via hydrothermal reaction at low temperature (Figure S1 shows the SEM image of the carbon cloth membrane with isotropic plain weave structure). Figure 1(a) shows a digital image of the dried rolled-up ZnO-NRs/CC membrane after a reaction time of 24 h. In this figure, the hydrothermal reaction changed the color from black for the original CC membrane to white for the ZnO-NRs/CC membrane. The inserted optical image in Fig. 1(a) is a top-view of the as-prepared ZnO-NRs/CC membranes showing that a uniform whitening of the membrane surface was obtained after 24 h hydrothermal reaction. In addition, the membrane retained its high flexibility even after the growth of the ZnO-NRs without altering its structural integrity indicating that this membrane can be applied in spiral wound format (Fig. 1(a)). Figure 1(b) shows that the carbon fibers of the carbon cloth membrane were wrapped thickly and uniformly by ZnO-NRs with a reaction duration of 24 h. More specifically, the nanorods grew vertically and intertwined each other all over the cylindrical surface of the each individual carbon fiber. Taking one single fiber at high magnification, the average length of the ZnO-NRs can be estimated by comparing the diameter of the original carbon fiber and the carbon fiber wrapped by the ZnO-NRs (Fig. 1(c)). The average length and diameter of the ZnO-NRs after the reaction time of 24 h were 6.3 ± 0.4 µm and 0.32 ± 0.09 µm, respectively. This average length of ZnO-NRs was closed to the average diameter of the carbon fiber that was 8.6 ± 0.5 µm. Figure 1(d) presents the XRD spectrums of the original CC membrane and the ZnO-NRs/CC membrane with 24 h reaction time. The detection of typical crystalline wurtzite structure of ZnO confirmed that the ZnO-NRs were successfully grown on CC membrane via hydrothermal reaction at low temperature^10,11^.

**Figure 2:**
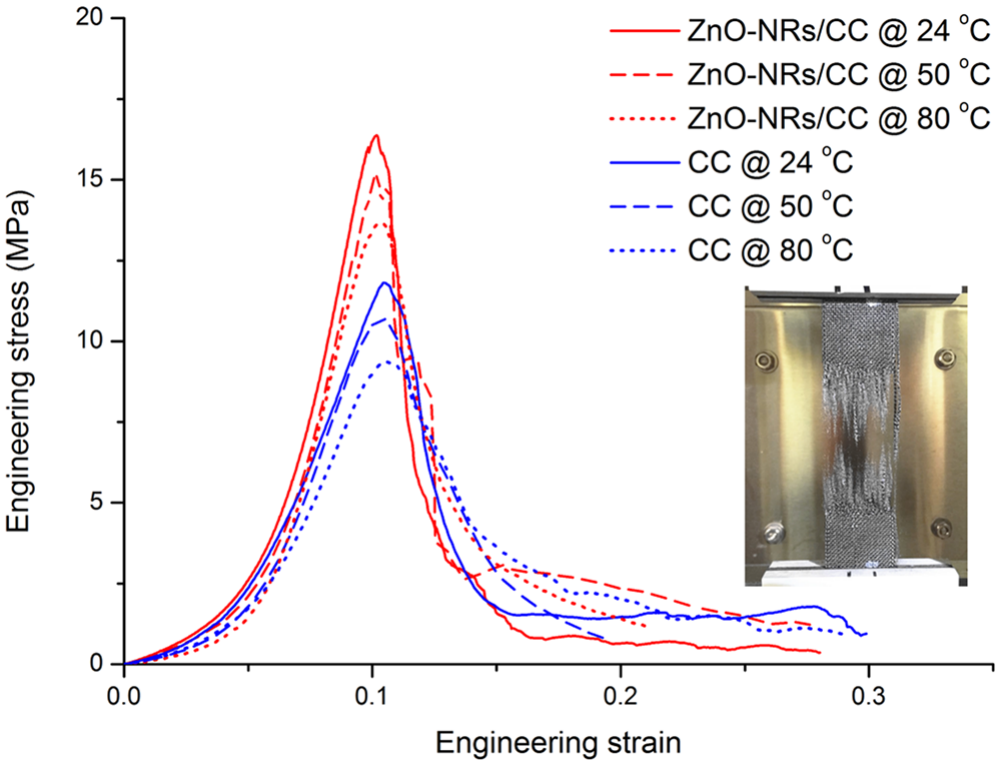
Engineering stress strain curves of CC and ZnO-NRs/CC membranes at different temperatures.

The uniaxial tensile properties of CC and ZnO-NRs/CC membranes at different temperatures are shown in Fig. 2. The ZnO-NRs/CC membranes were prepared by a reaction time of 24 h. The membranes were deformed till to the breakage. The corresponding mechanical parameters such as Young’s modulus and ultimate stress are listed in Table 1. In Fig. 2, the tensile behaviors of both CC and ZnO-NRs/CC membranes exhibited an initial non-linear stage for the strain ranging from 0 to 0.06, following by a linear elastic stage till to the ultimate stresses of the materials. After reached the ultimate stresses, the tensile stresses of the membranes sharply decreased with increasing strain. Compared with CC membranes, the mechanical responses of ZnO-NRs/CC membranes were higher for all temperatures. More specifically, the Young’s modulus increased from 168.4 MPa for CC membrane to 241.3 MPa for ZnO-NRs/CC membrane at 24 °C, representing an increase of 43.3%. While the ultimate stress increased from 11.4 MPa for CC membrane to 15.6 MPa for ZnO-NRs/CC membrane at same temperature, representing an increase of 36.8%. At 50 °C, the Young’s modulus and ultimate stress of ZnO-NRs/CC membrane had increases of 42.6% and 35.8%, respectively, compared to those of CC membrane. At 80 °C, the Young’s modulus increased from 149.0 MPa for CC membrane to 216.6 MPa for ZnO-NRs/CC membrane, representing an increase of 45.4%. And the ultimate stress increased from 9.4 MPa for CC membrane to 13.6 MPa for ZnO-NRs/CC membrane, representing an increase of 44.7%. In addition, unlike to polymeric materials whose mechanical behaviors are very sensitive to temperature^12^. The increasing temperature slightly decreased mechanical responses for both CC and ZnO-NRs membranes.

**Table 1:** Young’s modulus and ultimate stress of original CC and ZnO-NRs/CC membranes at different temperatures.

Membrane	Temperature (°C)	Young’s modulus (MPa)	Ultimate stress (MPa)
CC	24	168.4 ± 9.6	11.4 ± 0.4
	50	162.6 ± 10.6	10.9 ± 0.6
	80	149.0 ± 3.5	9.4 ± 0.9
ZnO-NRs/CC	24	241.3 ± 17.3	15.6 ± 0.7
	50	231.8 ± 4.2	14.8 ± 0.3
	80	216.6 ± 9.4	13.6 ± 0.4

The non-linearity of the stress-strain curves occurred at low applied stresses is due to the reorientation and straightening of yarns to the loading direction. Once the membranes reached the strain of 6% (Fig. 2), there was a further straightening and tension of the loaded yarn. The dominant deformation of the membranes is then mainly due to the uncramping and fiber stretching showed by higher slopes of the stress-strain curves^13,14^. Finally, carbon fibers in the tensile direction reach their deformation limit and the breakage occurs due to stress concentration, resulting in failures of the membranes as shown by the image of the sample in Fig. 2.

We note that the tensile behaviors of ZnO-NRs/CC membranes were higher than those of CC membranes at all temperatures. This is attributed to the growth of ZnO-NRs on the surface of CC membranes as well as inside CC membranes via hydrothermal reaction (Figure S2). The growth of ZnO-NRs formed a more dense porous structure of the ZnO-NRs/CC membranes. The interaction between the carbon fibers and ZnO-NRs benefited stress transfer resulting in higher mechanical properties. Note that the mechanical strength of ZnO-NRs/CC membranes are much higher than other polymeric membranes for water treatment^15–18^. More specifically, Zhu *et al*. fabricated a polyvinylidene fluoride (PVDF) based flat sheet membrane with a tensile stress of 7.75 MPa^15^. Hong *et al*. reinforced PVDF membrane by filling nano ZnO particles, and a maximum tensile strength of 2.92 MPa was measured when the Nano ZnO content was 0.01%^16^. Ma *et al*. prepared polysulfone (PSF) membranes with the addition of polyethylene glycol (PEG). In their study, a highest tensile stress of 5.62 MPa was found for the membrane with 8% of PEG. In addition, although the mechanical strength of ZnO-NRs/CC membranes are lower than other fragile ceramic based membranes^19–21^, the flexibility of ZnO-NRs/CC membranes allows them to be used in more practical applications. Moreover, the mechanical behaviors of ZnO-NRs/CC membranes with less temperature sensitivity allowed the membranes to be used in harsh conditions such as at high temperatures^22,23^.

**Figure 3:**
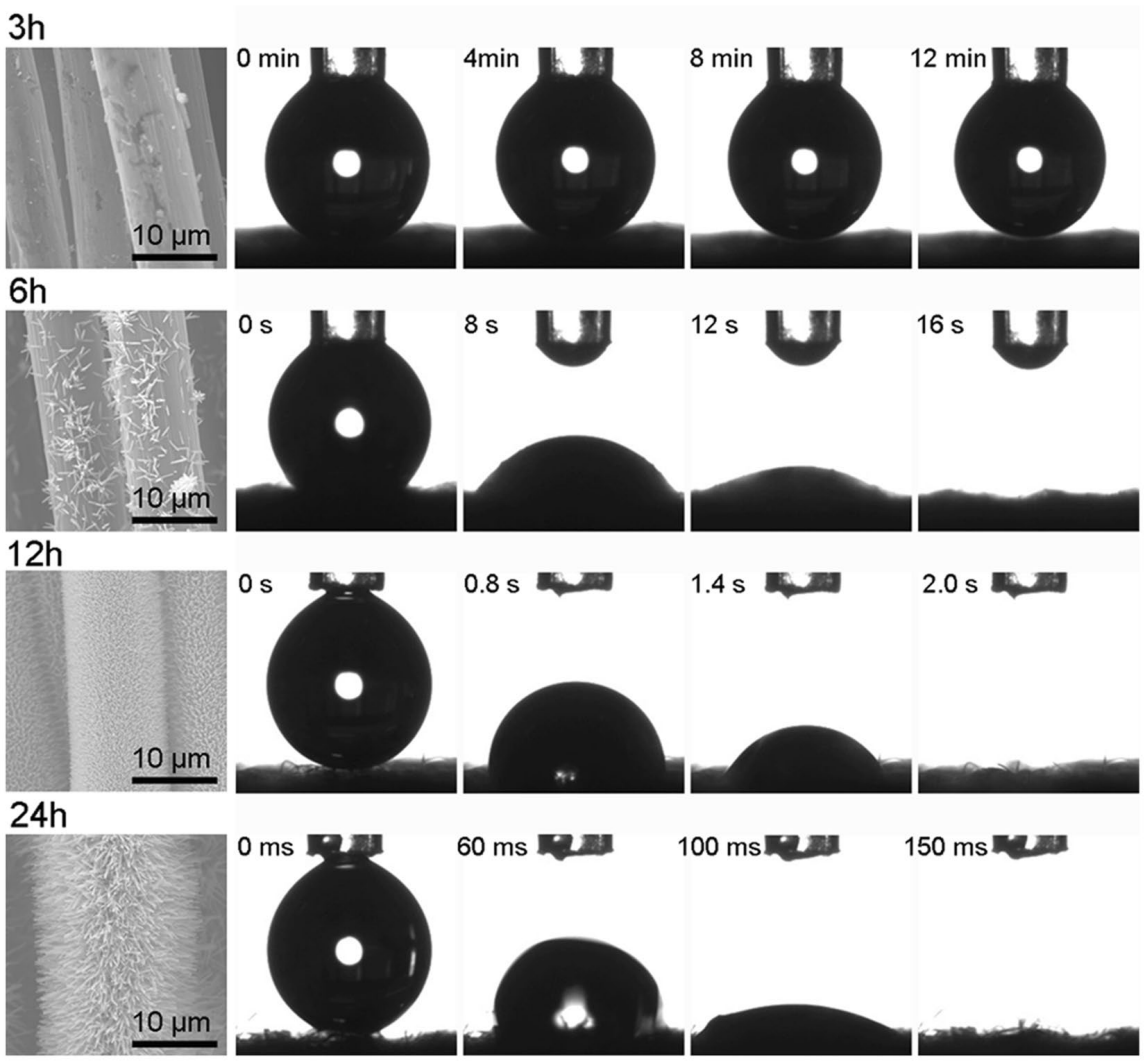
Time – dependent morphological effect on the wetting properties of ZnO-NRs/CC membranes.

The effect of hydrothermal reaction duration on the wetting behavior of the ZnO-NRs/CC membranes was examined by dynamic water spreading processes. Figure 3 shows the variation of spreading process of a water droplet into the membranes prepared with different reaction durations. The original activated CC membrane was hydrophobic exhibiting a water CA of about 150° (figure is not shown here). The water CA had not significant change for the membrane after 3 h reaction compared to the original CC membrane. After 3 h reaction, the water droplet spread slightly into the membrane after 12 min (Fig. 3). With increasing reaction time to 6 h, the water droplet spread out slowly into the membranes, and a zero CA was reached after 16 s. When the reaction time was 12 h, this spreading process was shortened to 2 s. A fastest spreading process of the water droplet into the membrane was recorded by the membrane with 24 h reaction time. This spread process was completed within 150 ms and then a zero water CA was obtained, showing an excellent superhydrophilicity of the membrane. Note that the wetting behaviors were also investigated for the membranes with the reaction time longer than 24 h. The spread processes of the water droplet into the ZnO-NRs/CC membranes with longer reaction duration were similar to that of the membrane prepared with 24 h reaction.

The wettability of the membranes can be related to the crystallographic morphology of the ZnO nanostructure that highly depends on the experimental parameters, such as reaction system, reaction temperature and solvent concentration^24–26^. In our study, the experimental results demonstrated that the hydrothermal reaction duration had a significant effect on the morphological properties of the ZnO-NRs/CC membranes, especially the density and length of the ZnO-NRs. In Fig. 3, few ZnO particles formed on the seeded carbon fibers after 3 h reaction time. When the reaction time increased from 3 h to 6 h, more particles formed and the structure of ZnO turned to rod-like particles. With increased reaction time to 12 h, the density of ZnO-NRs significantly increased and the carbon fibers were completely covered by ZnO-NRs. The most dense and longest ZnO-NRs grown on the carbon fiber surface were obtained at the reaction time of 24 h as shown in Fig. 3. With further increasing reaction time above 24 h, the ZnO-NRs became shorted but thicker. Similar observation was reported by Zhang *et al*.^27^ on a copper mesh based Cu(OH)_2_ nanowire membrane. In their study, the longest nanowires were found at a reaction time of 30 min. The nanowires became shorter and flower-bud like structures were formed with longer reaction time. Rivera *et al*.^28^ prepared high-aspect-ratio ZnO nanowires grown on a transparent conductive oxide surface by hydrothermal synthesis. It was found that the ZnO nanowires kept constant length but their diameter increased after a reaction time of 5 h, attributing to the dissolution of material from the polar nanowire tips and deposition onto the nonpolar side facets of the nanowires^28^. The time–dependent growth of ZnO-NRs was also investigated by EDS conducted on the ZnO-NRs/CC membrane surface (Figure S2). It can be seen that the weight percentage of zinc increased with increasing reaction time in our investigated range. This result is in agreement of our morphological observation (Fig. 3).

The superhydrophilicity of the ZnO-NRs/CC membrane with the reaction of 24 h was due to the hydrothermal reaction induced massive hydroxyl groups on the surface of the ZnO-NRs as well as the rough hieratical nanostructure of ZnO-NRs^29^. For a hydrophilic nano-scale rough surface with nanoporous structures, water prefers to fill the nanopores firstly due to a much larger capillary effect than the usual micro-scale pores^30,31^. In addition, the time-dependent growth of ZnO-NRs was not only on the surface but also inside of the CC substrate accelerating the spreading process of the water droplet into the membranes. The EDS investigations on the cross – section of ZnO-NRs/CC membrane indicated that the growth of ZnO-NRs inside the membranes increased with increasing reaction time up to 24 h (See Figure S2). Moreover, the water-favoring property of the ZnO-NRs/CC membranes was studied by analyzing their water capture ability. A highest water uptake amount of 169.8% was obtained for the ZnO-NRs/CC membrane with the reaction time of 24 h in our investigated range (Figure S5). The water capture abilities of the membranes are in agreement with their wetting behaviors (Fig. 3). Therefore, next sections will focus on the ZnO-NRs/CC membrane with the reaction time of 24 h.

**Figure 4:**
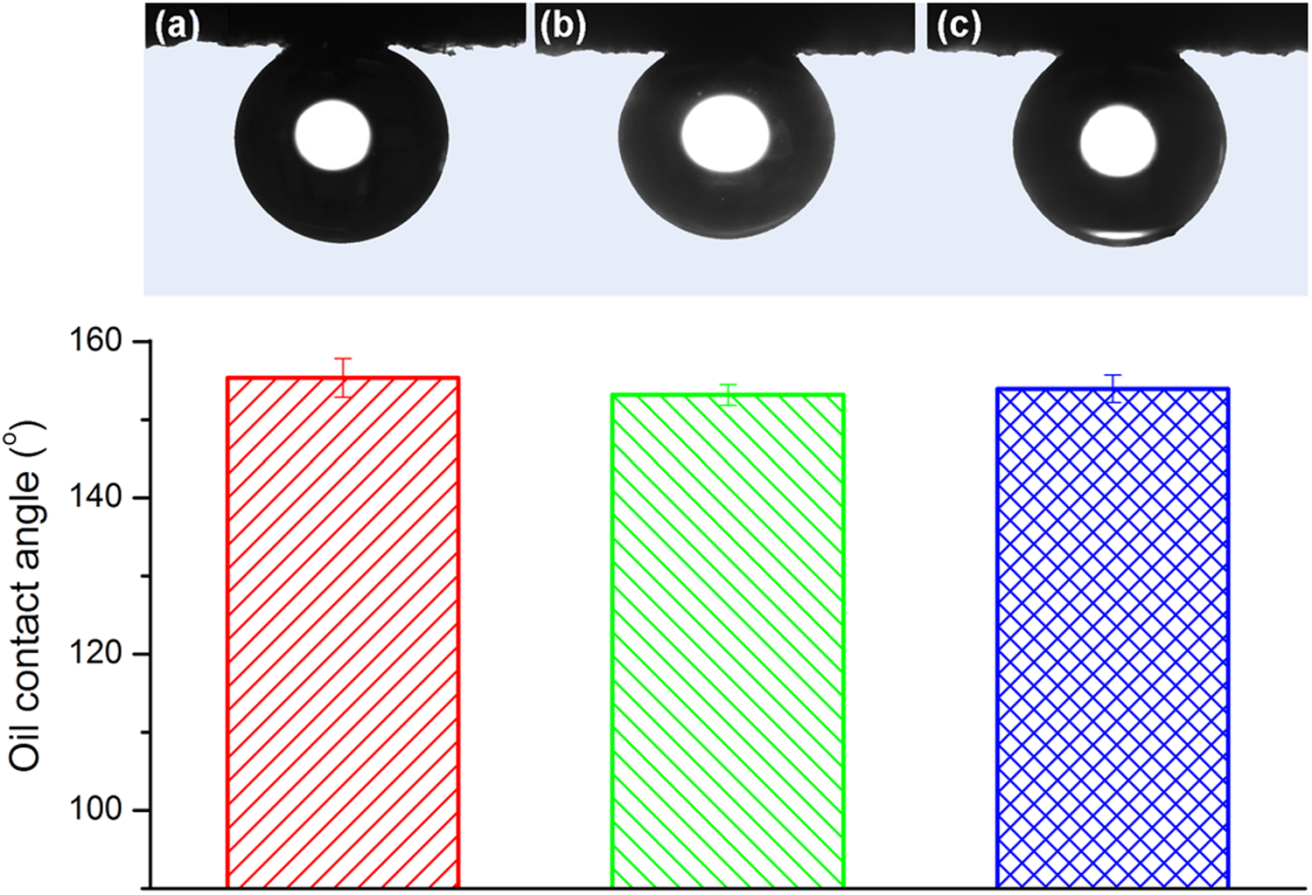
Underwater oil contact angles of (**a**) diesel, (**b**) gasoline and (**c**) sunflower oil for the ZnO-NRs/CC membrane with the hydrothermal reaction duration of 24 h.

The prepared ZnO-NRs/CC membranes with the hydrothermal reaction duration of 24 h showed excellent underwater superoleophobicity towards various oils, including diesel, gasoline and sunflower oil. Figure 4 shows the average underwater oil CAs of these three oils with the images of oil droplets on the membrane surface. As exhibited in Fig. 4, the underwater oil CAs of the three oils were all over 150°. More specifically, the oil CAs for diesel, gasoline and sunflower oil were 155.3° ± 2.5°, 153.2° ± 1.3° and 154.0° ± 1.8°, respectively. The underwater superoleophobicity of the membrane was attributed to their superhydrophilic surface as well as their rough hieratical nanostructure caused by the growth of the ZnO-NRs. Water molecules could be easily trapped in the interspaces of the rough surface to form a water cushion between the oil droplet and the solid membrane interface when the membrane was immersed into water. This formed interface between the oil/membrane can offer a strong repulsive force due to the repulsive interaction between polar water and non-polar oil molecules. In addition, the formed interface can also provide low oil-adhesion property of the membranes leading to less fouling probability by oil during the oil-water separation processes^32^. The solvent resistant properties of the ZnO-NRs/CC membranes with the hydrothermal reaction duration of 24 h was investigated by immersing the membranes in various organic solvents for 12 h. The underwater oil CAs was then measured. As exhibited in Figure S6, the membranes kept their underwater superoleophobicity after immersion into toluene, petroleum ether and hexane. The underwater oil CAs had no obvious change confirming the excellent solvent resistance property of the ZnO-NRs/CC membranes^27^.

**Figure 5:**
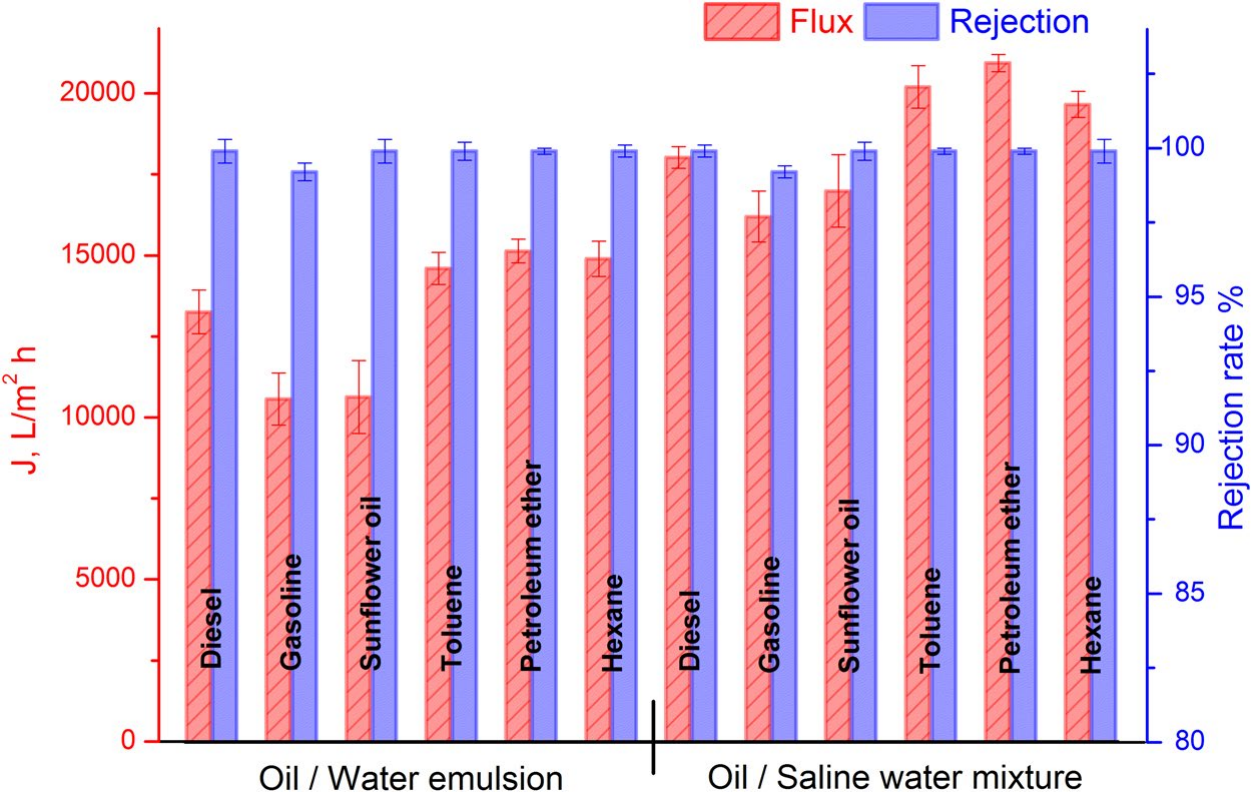
Water permeation fluxes and rejection rates of the gravity-driven separation process using ZnO-NRs/CC membrane for oil-in-water emulsions and for oil-in-saline-water mixtures containing diesel, gasoline, sunflower oil, toluene, petroleum ether and hexane.

The superhydrophilicity, surperwettability and underwater superoleophobicity enabled the ZnO-NRs/CC membranes to get great potential in treatment of oil-containing wastewater. During the gravity-driven separation process, the wetting phase of water quickly passed through the membranes and the oil was retained above the membranes. As shown in Fig. 5, all the six oil-in-water emulsions and six oil-in-saline-water mixtures exhibited promising high fluxes. The highest flux of 20933.4 L m^−2^ h^−1^ was obtained for petroleum-ether-in-saline-water mixtures. Even though the sunflower-oil-in-water emulsions had a lowest permeation flux of 10639.2 L m^−2^ h^−1^ in the current study, this flux is still much higher than other gravity-driven oil-in-water separation membranes^32–35^. The gravity-driven membranes with high fluxes are very attractive from the viewpoint of energy conservation compared to the traditional pressure-driven ultrafiltration membranes for the application of oil-water separation. In addition, the higher permeation fluxes of oil-in-saline-water mixtures compared to those of oil-in-water emulsions are mainly due to their lower viscosities. Basically, an oil-in-water mixture with a lower oil viscosity exhibits higher flux^7^.

**Figure 6:**
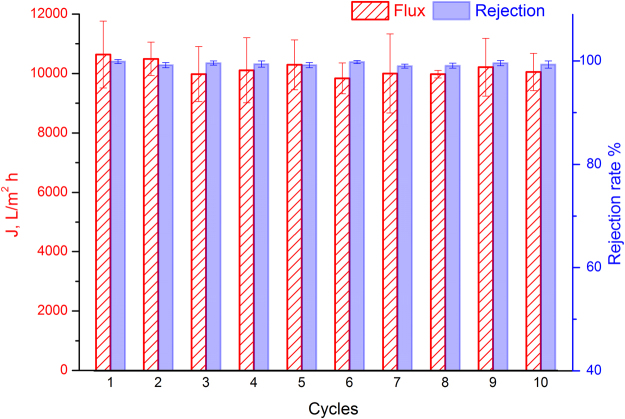
Water permeation fluxes and rejection rates of sunflower-oil-in-water emulsions separated by the ZnO-NRs/CC membrane as a function of repeated separation processes. In one cycle, a certain emulsion permeated the membrane first and the membrane was then washed by passing hot water.

Figure S7 demonstrates oil droplet size distribution in as-prepared oil-in-water emulsions. It can be seen that the oil droplet size mainly ranged from 5 to 25 μm. The average oil particle size of the oil-in-water emulsions was 16.6 μm. Concerning the filtrate qualities, the separation efficiencies were all above 99% for all oil-in-water emulsions and oil-in-saline-water mixtures as shown in Fig. 5. To further see the separation effect, optical microscopy was used to observe the difference between the original emulsions and the collected filtrates. As shown in Figure S8, no visible oil droplets were observed in the corresponding filtrate in our investigated range, showing an excellent separation efficiency. The high separation efficiencies are mainly due to the superhydrophilicity, superwettability and underwater superoleophobicity of the ZnO-NRs/CC membrane with the hydrothermal reaction of 24 h. In addition, our membranes can be easily cleaned by rinsing. More specifically, ZnO-NRs/CC membrane was washed by passing hot water after oil-in-water emulsion separation process. It was found that the water fluxes remained stable (Fig. 6), and the rejection rates were all over 99% with increasing cycle times up to 10. These results prove that our ZnO-NRs/CC membrane features excellent antifouling property and stability for oil-in-water emulsion separation, due to its superhydrophilicity and underwater superoleophobicity characteristics. Moreover, the robustness of the ZnO-NRs/CC membrane was studied by the abrasion resistant test. To this end, the membranes with a hydrothermal reaction time of 24 h was dragged facing the surface of a 1500 grit sandpaper with a distance of 10 cm. A load of 100 g was vertically applied by putting a weight on the membranes^36^. Figure S9 shows the dynamic water spreading behavior of the ZnO-NRs/CC membranes as a function of abrasion test cycle. In this Figure, it can be seen that the water spreading time increased with increasing abrasion test cycles. After twenty times abrasion test for the membrane, the water droplet spread out into the membrane within 3.3 s. Although the spreading process for the membrane with 20 cycle abrasion was much slower compared with the new membrane (0 cycle), the water spreading time was still shorter than the new membrane with 6 h reaction as shown in Fig. 3, indicating that superhydrophilicity of the abraded membrane still remains. This maintaining of the water spreading behavior after harsh abrasion proved the robustness of the ZnO-NRs/CC membrane.

## Conclusions

In this work, a commercial carbon cloth (CC) based all-inorganic membrane was prepared by the growth of three-dimensional ZnO nanorod arrays (NRs) via hydrothermal route at low temperature. The time-dependent ZnO growth process showed that the carbon cloth fibers were wrapped thickly and uniformly by long ZnO-NRs with a reaction duration of 24 h. Due to the hydrothermal reaction induced massive hydroxyl groups on the surface of the ZnO-NRs as well as the formation of rough hieratical nanostructure of ZnO-NRs, the prepared ZnO-NRs/CC membranes exhibited superhydrophilicity and superwettability, showing a ultrafast spreading process of the water droplet into the membrane within 150 ms and a water capture ability of 169.8%. The superhydrophilicity of the membrane facilitated the formation of a water cushion between the oil droplet and the solid membrane interface resulting in underwater superoleophobic property of the membrane. Driven by gravity solely, the prepared ZnO-NRs/CC membranes can separate both oil-in-water emulsions with average oil particle size of 16.6 µm and oil-in-saline-water mixtures. The separation rate were all over 99% and the water permeation flux was as high as 20933.4 L m^−2^ h^−1^, which were among the best values reported. Beside excellent separation properties, the prepared ZnO-NRs/CC exhibited good mechanical strength and flexibility. The growth of ZnO-NRs resulted in significantly increased Young’s modulus and the ultimate tensile strength compared to the original CC membrane at room temperature as well as at high temperatures. This increase is attributed to the growth of ZnO-NRs on the surface of CC membranes as well as inside CC membranes. The interaction between the carbon fibers and ZnO-NRs benefited stress transfer resulting in higher mechanical properties of the membranes.

## Methods

### Materials

A commercial carbon cloth with plain weave structure referenced as Carbon Cloth CC4 Plain from Fuel cell Earth (Woburn, MA, USA) was used in this study. Chemically pure zinc acetate was purchased from Surechem Products LTD. (Suffolk, UK). Ammonia (28%, analytical grade) and potassium hydroxide were supplied by VWR International (Radnor, PA, USA).

### Preparation of carbon cloth substrate*s*

Before the preparation of ZnO/carbon cloth membranes, the as received carbon cloth was cut into a suitable dimension and then activated by sonication sequentially in acetone, deionized (DI) water, and ethanol for 30 min each^7^. After the activation, the carbon cloth substrates were dried in an oven for 1 h at 95 °C and then cooled at room temperature.

### Preparation of seed layer for ZnO nanorod*s*

A seed solution was prepared by sol–gel method. More specifically, an ethanilic solution of zinc acetate was first prepared (0.01 mol/L in 125 mL), and then another ethanilic solution of potassium hydroxide was dropped (0.03 mol/L in 65 mL). The mixture of the two ethanilic solutions were magnetically stirred at 60 °C for 2 h to obtain a transparent and homogeneous seed solution. The carbon cloth substrates were immersed into the prepared seed solution and then dried at 95 °C for 30 min by using an oven. The immersion of the carbon cloth substrates into the seed solution was repeated three times in order to obtain a uniform seed layer on the substrates^37^.

### Growth of ZnO Nanorods

The solution for ZnO nanorods growth was prepared by dissolving zinc acetate (0.005 mol/L) in DI water (95 mL). 5 mL of ammonia solution was dropped into the zinc acetate solution mixing by a magnetic stirrer. The prepared solution for the growth of ZnO nanorods was then transferred into sealable glass beakers. The carbon cloth substrates were suspended into the sealable glass beakers within the solution and kept into an oven at 95 °C. In the following, carbon cloth and ZnO nanorods/carbon cloth membranes were denoted as CC and ZnO-NRs/CC membranes, respectively.

### Preparation of oil-in-water emulsions and oil-in-saline water mixtures

The oil-in-water emulsions were prepared by mixing water and oil (diesel, gasoline, sunflower oil, toluene, petroleum ether and hexane) in a volume ratio of 9:1 v/v and rotated by Vortex mixer (VELP Scientifica Srl, Usmate, Italy) at a speed of 3000 rmp for 30 s to obtain a homogeneous milky solution. Note that the prepared oil-in-water emulsions could be stable for at least 60 min under ambient conditions. In order to expand the applications of our membranes in seawater environment, salt was firstly dissolved in DI water (35 g/L) to obtain the saline water. The oil-in-saline water mixtures were then prepared by mixing the saline water and oil (diesel, gasoline, sunflower oil, toluene, petroleum ether and hexane) in a volume ratio of 9:1 v/v with strong shaking.

### Characterization

A FEI Quanta 400 (Hillsboro, OR, USA) environmental scanning electron microscope (ESEM) equipped with EDAX Apollo Energy Dispersive X-ray Spectroscopy (EDS) system, was used for studying the morphological properties of the prepared membranes. For SEM analysis, the samples were coated with gold using Leica EM SCD050 coating machine. X-ray diffraction (XRD) experiments of the prepared membranes were carried out using a Rigaku Ultima IV multipurpose X-ray diffractometer (Tokyo, Japan) equipped with Cu Ka radiation and fixed monochromator. An acceleration voltage of 40 kV and 20 mA current were applied. The XRD patterns were collected using a continuous scan mode and 2theta (2θ) angle with sampling width of 0.02 degree and scan speed of 1 degree/minute. The hydrophilicity and under-water oleophobicity of the membranes were studied using an advanced goniometer (Rame-hart model 500, Succasunna, NJ, USA). The hydrophilicity of the membranes was investigated by analyzing the spreading process of the water into the membranes. To this end, a water droplet of 3 µL was dispensed onto the membranes surface in air. A high-speed camera with a speed of 100 frames/second was used to record the spreading process. For under-water oil contact angle (CA) measurement, the membranes were first immersed in DI water. Oil droplets of about 14 µL were then dropped onto the membranes from the bottom, and the under-water oil CAs were measured. Each measurement was repeated on 3 samples, we report in this paper the averaged values. A filtration device (Nalgene 300–4050, Rochester, NY, USA) with an effective membrane area of 11.3 cm^2^ was used for evaluating the separation properties of the fabricated membranes. A 100 mL amount of the prepared oil-in-water emulsions or oil-in-saline water mixtures was poured into the filtration device. The separation process was purely driven by gravity. The filtrate was collected and the oil content in the water was detected using a Shimadzu total organic carbon (TOC) analyzer (Nakagyo-ku, Kyoto, Japan) to examine the separation efficiency. The membrane water flux was calculated by measuring the time needed to collect some volume of permeate, which pass through the membrane. Uniaxial tensile properties of the ZnO-NRs/CC membranes with a gauge length of 50 mm and a width of 10 mm were investigated by means of a MTS Insight universal test system (Eden Prairie, MN, USA). The jaws for holding the membranes were equipped with a rubberized surface to avoid sample sliding. Tensile tests were conducted at two different temperatures of 24 °C (room temperature) and 50 °C. To get the same experimental condition for each test, at each change of temperature, the environmental chamber was first heated without specimen for 30 min to obtain a uniform temperature. Then, the specimen was placed in the chamber for 10 min to allow thermal equilibration, and then was tested. The Young’s modulus and ultimate stress of the membranes were assessed at a crosshead speed of 5 mm/min. Five tests were performed per material (CC and ZnO-NRs/CC) to obtain averaged values of each mechanical parameter.

## Electronic supplementary material

Supplemetary Information
