# Supplementary material for: A windable and stretchable three-dimensional all-inorganic membrane for efficient oil/water separation
Source: Sci Rep. 2017 Nov 22;7:16081. doi: 10.1038/s41598-017-16402-5 (PMC5700037; doi:10.1038/s41598-017-16402-5)
Supplement: Supplementary file 1 — Supplemetary Information [file 41598_2017_16402_MOESM1_ESM.pdf]

Supporting Information:

A windable and stretchable three-dimensional all-inorganic membrane for efficient oil/water separation

Kui Wang[1,2], Dong Suk Han[3], Wubulikasimu Yiming[3], Said Ahzi[1], Ahmed Abdel-Wahab[3], Zhaoyang Liu[1*]

[1]Qatar Environment and Energy Research Institute (QEERI), Hamad Bin Khalifa University (HBKU), Qatar Foundation, PO Box 5825, Doha, Qatar

[2]Key Laboratory of Traffic Safety on Track of Ministry of Education, School of Traffic & Transportation Engineering, Central South University, Changsha 410075, China

[3]Chemical Engineering Program, Texas A&M University at Qatar, Education City, Doha 23874, Qatar

* Corresponding author: E-mail address: zhliu@qf.org.qa (Zhaoyang Liu).

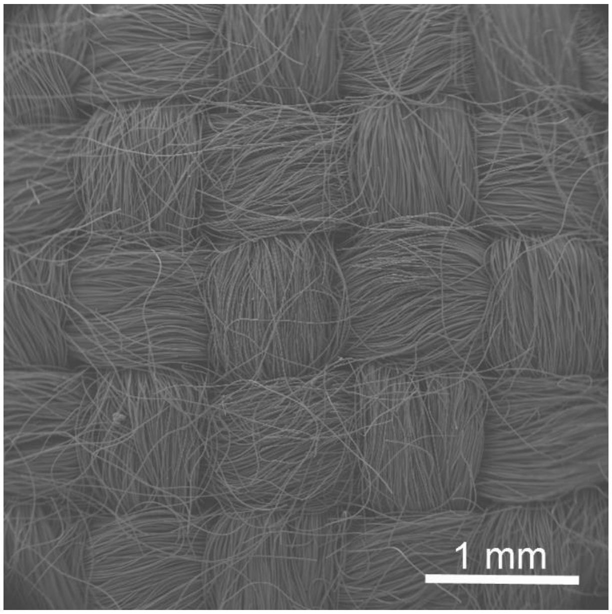

Figure S1: SEM image of the carbon cloth CC4 Plain membrane with isotropic plain weave structure.

Figure S2 (a) exhibits the cross-section SEM images of the ZnO-NRs / CC membranes with a hydrothermal reaction time of 24 h for growing ZnO-NRs on CC membrane. As shown in Figure S2 (a), the time-dependent growth of ZnO-NRs was not only on the surface but also inside of the CC substrate, attributing to the effective hydrothermal reaction. Figures S2 (b) and (c) show EDS analysis of the elemental composition on the surface and cross-section of ZnO-NRs / CC membranes for different hydrothermal reaction durations. The insets are corresponded EDS spectrums of the membranes with the hydrothermal reaction duration of 24 h. In Figures S2 (b) and (c), it can be seen that the weight percentage of zinc on both membrane surface and cross-section increased with increasing reaction time. The main elements detected on the membrane surface and cross-section were Zn and O with the reaction time of 24 h, illustrating the successful growth of ZnO on membrane surface as well as inside membrane. This result is also in agreement of our X-ray photoelectron spectroscopy characterization of the membranes (Table S1), where the intensity of Zn and O on ZnO-NRs / CC membrane increased with increasing reaction duration.

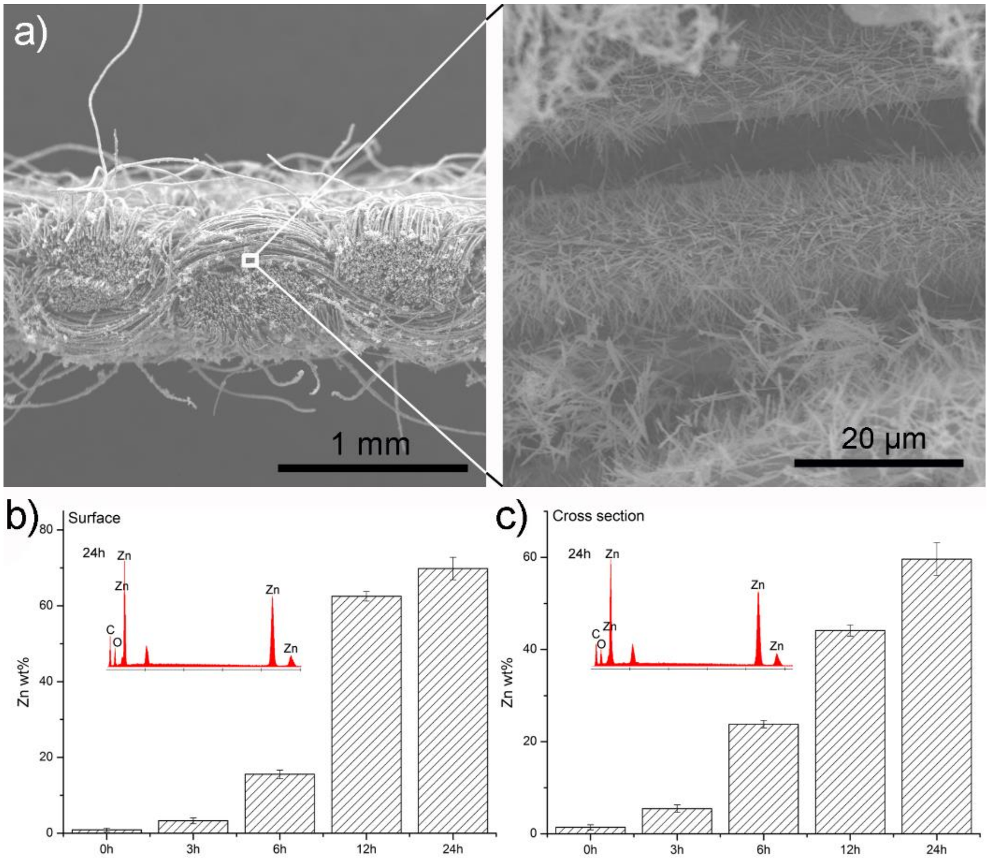

Figure S2: (a) Cross-section SEM images of the ZnO-NRs / CC membranes with a hydrothermal reaction time of 24 h for growing ZnO-NRs on CC membrane. EDS analysis of the elemental composition on the (b) surface and (c) cross-section of ZnO-NRs / CC membranes for different hydrothermal reaction durations.

The element composition of CC and ZnO-NRs / CC membranes were analyzed using X-ray photoelectron spectroscopy (XPS, Kratos AXIS Ultra) with a monochromatic Al Kα X-ray source (hν=1486.6 eV) and a delay-line detector (DLD). A wide range of binding energy (eV) was applied from 1200 to 0 with pass energy of 80 eV and with 2 sweeps in order to identify all elemental information. In addition, elemental mapping of Zn and C was collected from 1012.8 eV of Zn 2p and 284.6 eV of C 1s, respectively, by using non-monochromatic a dual anode Mg Kα (1.253 KeV) source. Figure S3 shows that the intensity of Zn peak increased with increasing hydrothermal reaction time from 3h to 24h. The quantified element percentage obtained by XPS

is exhibited in Table S1. Although there was no significant increased amount of Zn element between 3h and 6h reaction time, the amount of Zn element for membrane with 12h reaction time substantially increased by a factor of 6.7 as compared to 6h membrane. Furthermore, the Zn amounts for the membrane with 24h reaction duration increased by 1.6 times compared to 12h membrane. In addition, in the elemental XPS mapping image (Figure S4), the ZnO-NRs / CC membrane with 24 hours of hydrothermal reaction time shows well ripening of ZnO-NRs (green color) alongside stripes of carbon cloth compared to others.

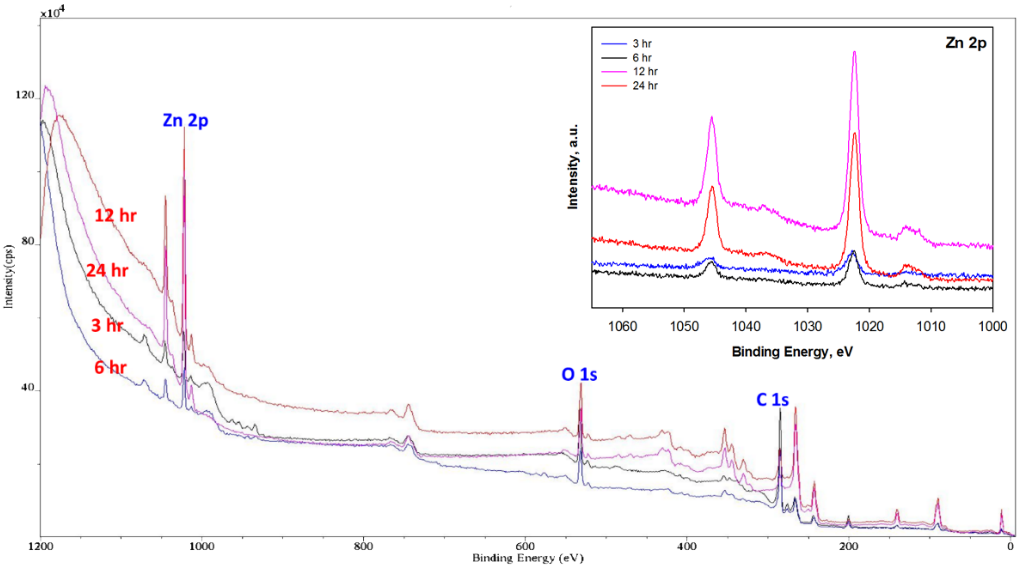

Figure S3. A wide survey XPS spectra of the ZnO-NRs grown on the CC membrane as functions of hydrothermal reaction time.

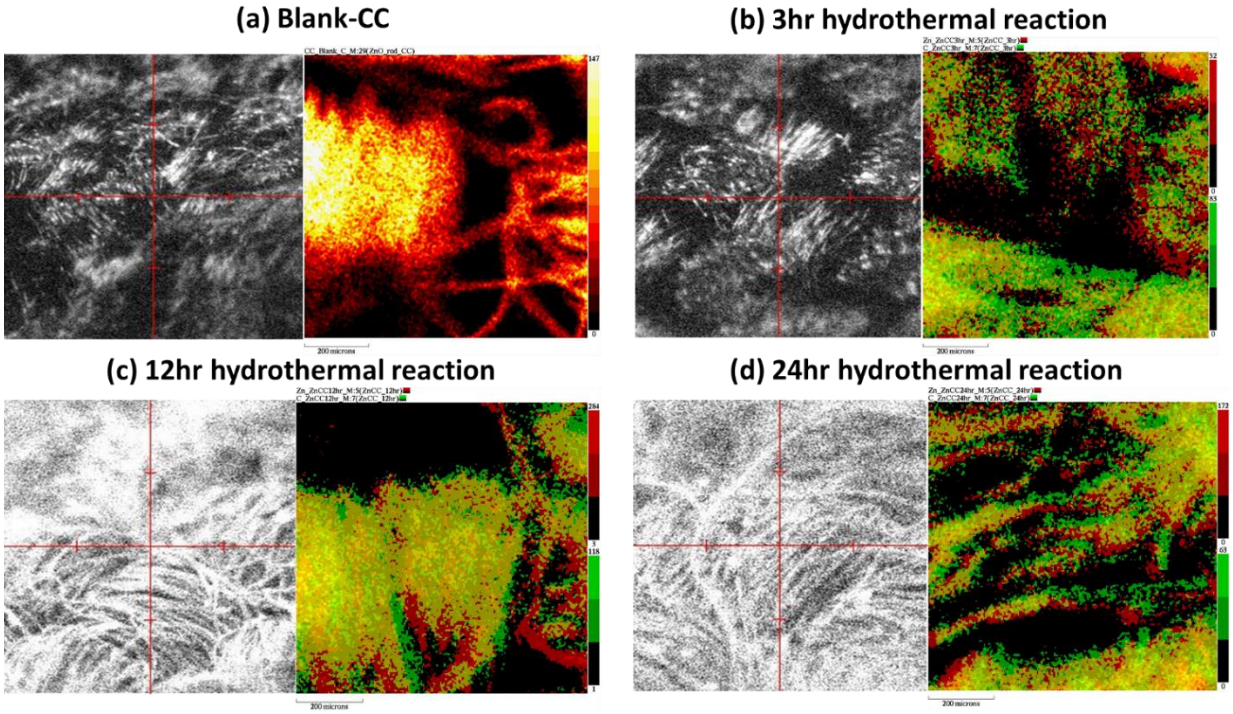

Figure S4. Elemental XPS mapping of ZnO-NRs grown on the carbon cloth as functions of hydrothermal reaction time.

Table S1. Quantified element amount as a function of reaction time obtained from wide survey XPS spectra.

Element (%)	3 h	6 h	12 h	24 h
Zn 2p	2.60	2.74	18.43	28.94
C 1s	17.84	19.31	31.00	41.49
O 1s	79.56	77.94	50.57	29.57

The growth of ZnO-NRs via hydrothermal reaction changed CC membrane from hydrophobicity to superhydrophilicity. To prove the water-favoring property of ZnO-NRs / CC membranes, water capture ability (WCA) was measured for the membranes with different reaction durations using following equation:

$$WCA = \frac{W_b - W_a}{W_a} \times 100\%$$

here, W_a is the original mass of the membrane before water immersion. W_b is the mass of the membrane after immersed in water for 10 s and then quickly taken out from water. Figure S5 shows the water capture percentage of the ZnO-NRs / CC membranes as a function of hydrothermal reaction duration. It can be found that the seeded CC membrane (0 h) was hydrophobic having a small amount of water uptake about 26.0 %. With 3 h reaction, the WCA of the ZnO-NRs / CC membrane had no significant change compared to that of the original seeded CC membrane. However, increasing the reaction time from 3 h to 6 h, the water capture amount of the membrane sharply increased to 121.7 %. With further increasing reaction time from 6 h to 12 h, the water capture percentage of the membrane slightly increased to 157.5 %. A highest water uptake amount of 169.8 % was obtained for the ZnO-NRs / CC membrane with the reaction time of 24 h in our investigated range. Similar to the wettability of ZnO-NRs / CC membranes shown in Figure 3. The WCA of the ZnO-NRs / CC membranes can also be related to the time – dependent growth of the ZnO-NRs on the CC membranes via hydrothermal reaction. The increased water capture percentage of the ZnO-NRs / CC membranes with increasing reaction time confirmed that the hydrothermal reaction is an effective method to prepare superhydrophilicity ZnO-NRs / CC membranes with hieratical nanostructure.

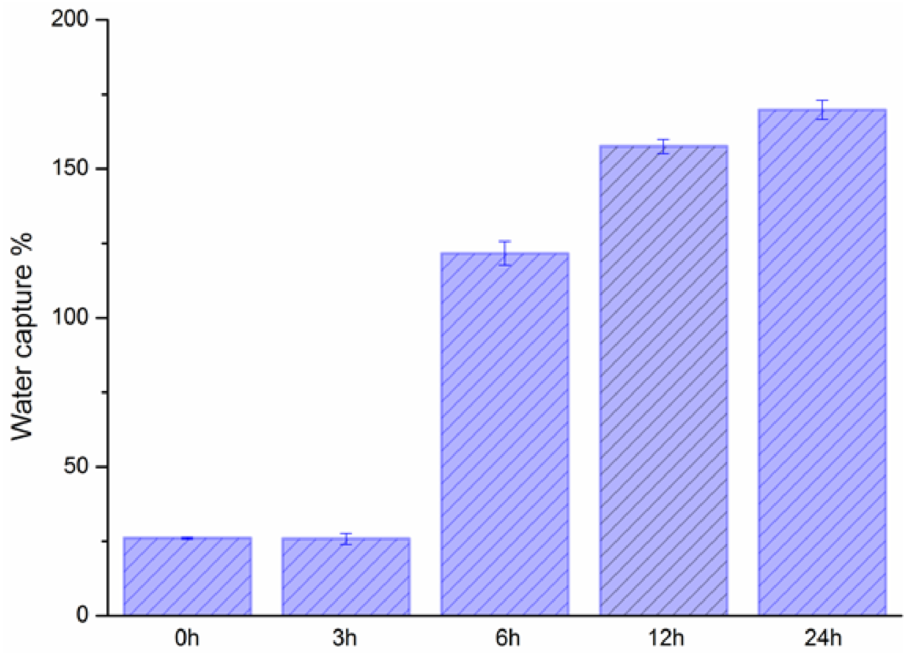

Figure S5: Water capture ability of the ZnO-NRs / CC membranes as a function of hydrothermal reaction duration.

As shown in Figure S6, the ZnO-NRs / CC membranes kept their underwater superoleophobicity after immersion into toluene, petroleum ether and hexane for 12h. The underwater oil CAs had no obvious change confirming the excellent solvent resistance property of the ZnO-NRs / CC membranes.

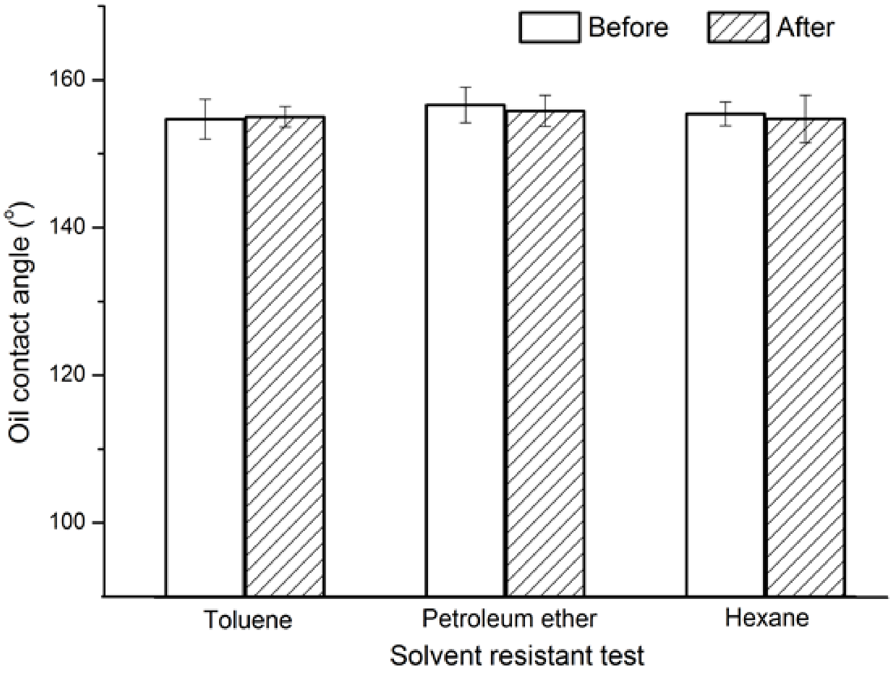

Figure S6: The underwater oil CAs of the ZnO-NRs / CC membranes with the hydrothermal reaction duration of 24 h before and after immersing in various organic solvents for 12 h.

The droplet sizes of the oil-in-water emulsions were measured by an Olympus IX73 (Shinjuku, Tokyo, Japan) optical microscopy. This equipment was equipped with a digital camera coupled with an Olympus cellSens acquisition software. The oil particle size was analyzed with the software Image-J using the recorded optical images (National Institutes of Health, Maryland, USA). Figure S7 exhibits diesel particle size distribution for sunflower oil-in-water emulsions. The statistical histogram in Figure S7 shows a narrow distribution of diesel droplet size mainly ranging from 5 to 25 μm. The average oil particle size of the diesel-in-water emulsions was 16.6 μm. The narrow distribution of the oil droplets size and the small average droplet diameter indicated that our preparation for oil-in-water emulsions was effective and controllable. Note that the oil particle size distribution and average oil particle size for other gasoline-in-water emulsions and sunflower-oil-in-water emulsions had similar results.

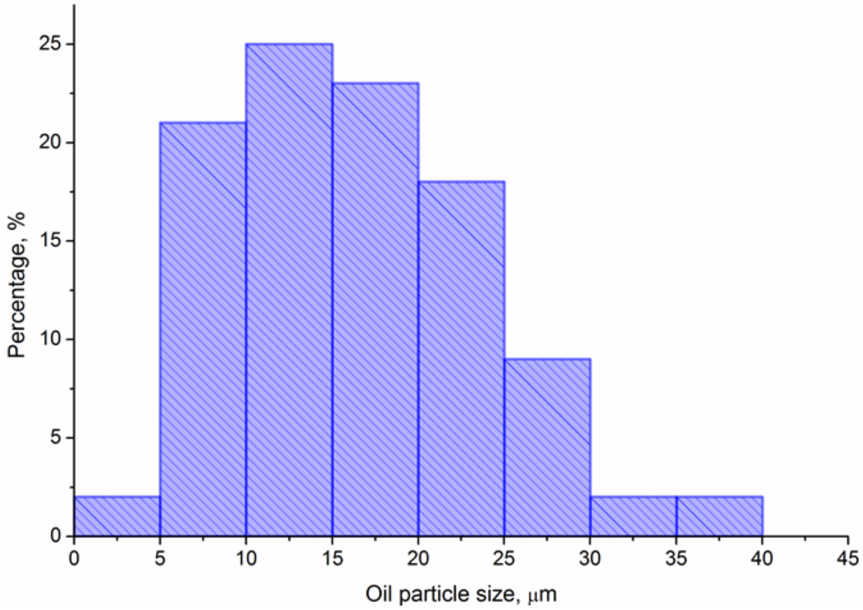

Figure S7: Diesel particle size distribution in as-prepared oil-in-water emulsions

Digital image of as-prepared milky diesel-in-water emulsions is shown in Figure S8 (a). Dense oil droplets in micrometer-scale could be clearly observed in the corresponded optical microscopic image. After the gravity-driven separation process using ZnO-NRs / CC membrane with the reaction time of 24 h, the collected filtrate had a significant change in color and light transmission as displayed in Figure S8 (b). No visible oil droplets were observed in the corresponded filtrate using optical microscope, indicating an excellent separation efficiency.

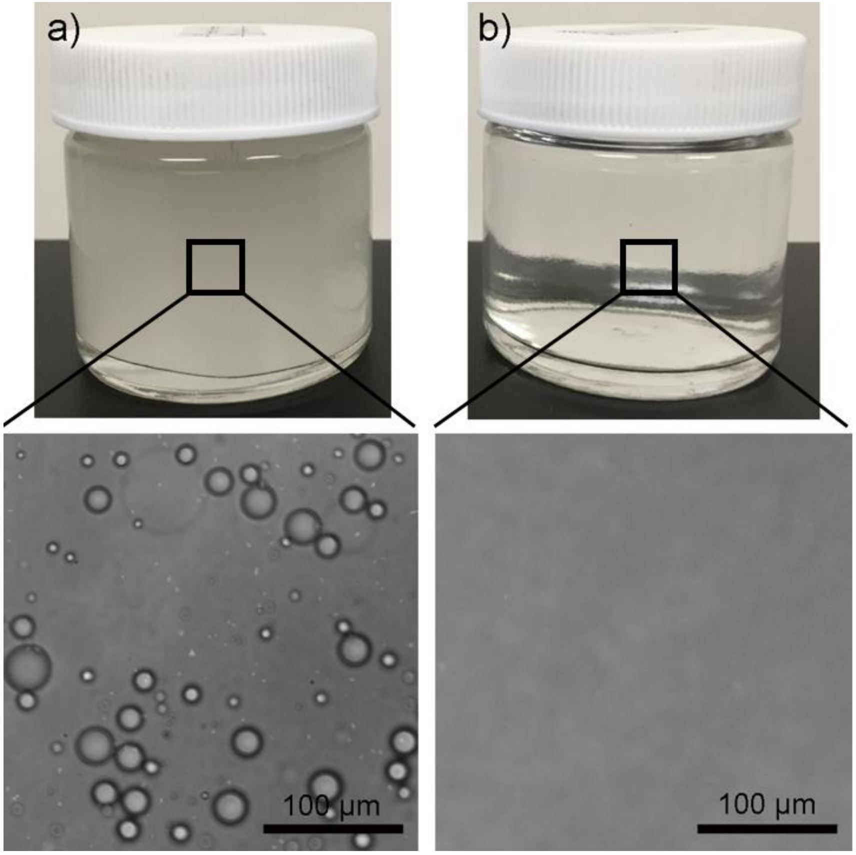

Figure S8: Photos and microscope images for (a) As-prepared diesel-in-water emulsions (b) permeation of diesel-in-water emulsions separated using Zn-NRs / CC membrane with a reaction time of 24 h.

Figure S9 shows the dynamic water spreading behavior of the ZnO-NRs / CC membranes as a function of abrasion test cycle. For the abrasion test, the membranes with a hydrothermal reaction time of 24h was dragged facing the surface of a 1500 grit sandpaper with a moving distance of 10 cm. A load of 100g was vertically applied by putting a weight on the membranes. After twenty times abrasion cycles for the membrane, the water droplet spread out into the membrane within 3.3 s, indicating superhydrophilicity of the abraded membrane. This maintaining of the water spreading behavior after harsh abrasion proved the robustness of the ZnO-NRs / CC membrane.

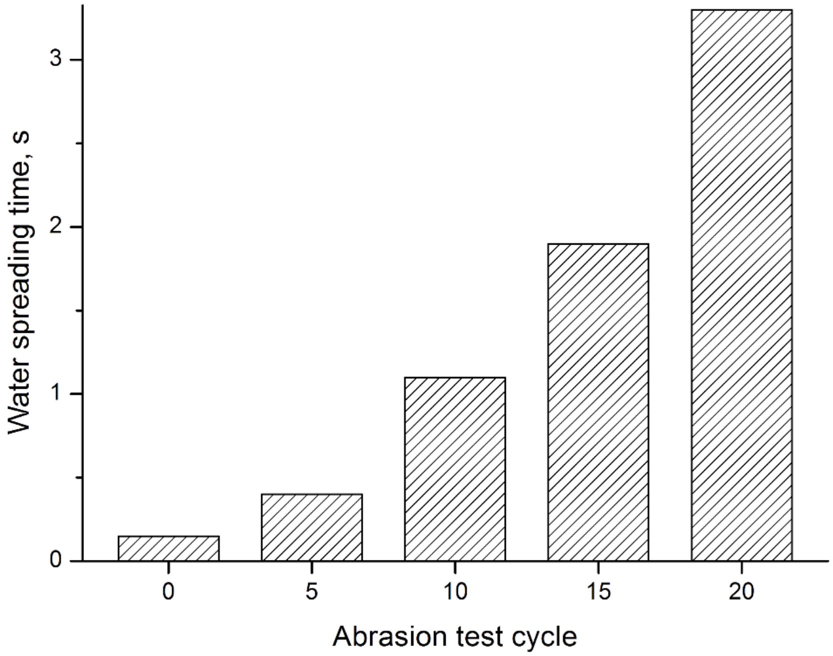

Figure S9: Water spreading behavior of the ZnO-NRs / CC membranes as a function of abrasion test cycle.
